# Finding safety: a pilot study of managed alcohol program participants’ perceptions of housing and quality of life

**DOI:** 10.1186/s12954-016-0102-5

**Published:** 2016-05-09

**Authors:** Bernadette (Bernie) Pauly, Erin Gray, Kathleen Perkin, Clifton Chow, Kate Vallance, Bonnie Krysowaty, Timothy Stockwell

**Affiliations:** Scientist Centre for Addictions Research of BC, University of Victoria, Victoria, BC Canada; School of Social Work, MacEwan University, Edmonton, Canada; Centre for Addictions Research of BC, Victoria, Canada

**Keywords:** Severe alcohol use, Alcohol dependency, Homelessness, Housing first, Managed alcohol programs, Harm reduction, Alcohol harm reduction

## Abstract

**Background:**

There is a higher prevalence of alcohol use and severe alcohol dependence among homeless populations. The combination of alcohol use and lack of housing contributes to increased vulnerability to the harms of substance use including stigma, injury, illness, and death. Managed alcohol programs (MAPs) administer prescribed doses of alcohol at regular intervals to people with severe and chronic alcohol dependence and homelessness. As a pilot for a larger national study of MAPs, we conducted an in-depth evaluation of one program in Ontario, Canada. In this paper, we report on housing and quality of life outcomes and experiences of the MAP participants and staff.

**Methods:**

We conducted a pilot study using mixed methods. The sample consisted of 38 people enrolled in or eligible for entry into a MAP who completed a structured quantitative survey that included measures related to their housing and quality of life. All of the participants self-identified as Indigenous. In addition, we conducted 11 in-depth qualitative interviews with seven MAP residents and four program staff and analyzed the interviews using constant comparative analysis. The qualitative analysis was informed by Rhodes’ risk environment framework.

**Results:**

When compared to controls, MAP participants were more likely to retain their housing and experienced increased safety and improved quality of life compared to life on the streets, in jails, shelters, or hospitals. They described the MAP as a safe place characterized by caring, respect, trust and a nonjudgmental approach with a sense of family and home as well as opportunities to reconnect with family members.

**Conclusions:**

The MAP was, as described by participants, a safer environment and a home with feelings of family and a sense of community that countered stigma, loss, and dislocation with potential for healing and recovery. The MAP environment characterized by caring, respect, trust, a sense of home, “feeling like family”, and the opportunities for family and cultural reconnections is consistent with First Nations principles for healing and recovery and principles of harm reduction.

Among homeless populations, the rate of severe alcohol dependence is higher than in the general population for both men and women [1, 2]. The use and related harms of “illicit” or nonbeverage alcohol such as rubbing alcohol, hand sanitizer, and mouthwash have been identified as concerns among homeless populations and can act as a barrier to obtaining housing [3–5]. Severe alcohol dependence almost invariably carries heavy health and social costs [6–8] including a range of acute, chronic, and social harms. For people living in socially marginalizing conditions, the combination of severe alcohol use and lack of housing contributes to increased vulnerability to harms such as stigma, freezing, violence, accidents, physical illness, and death.

Managed alcohol programs (MAPs) are a harm reduction strategy that incorporates the provision of regulated doses of alcohol alongside accommodation and other supports to address the twin harms of severe alcohol dependence and homelessness. MAPs, in Canada, have developed as a compassionate response to the pressing and complex needs of a group for whom structural conditions such as homelessness, low income, and trauma contribute to poor health, high mortality, increased health services, and policing costs as well as difficulties finding and keeping housing. As a pilot for a larger national study of MAPs, we conducted an in-depth evaluation of one program in Thunder Bay, Ontario, Canada. In this paper, we report specifically on housing and quality of life outcomes as well as participant perceptions of the role of the MAP program in their lives. A companion paper (Vallance et al., in press) reports on outcomes relating to pattern of alcohol consumption and related harms.

## Background

Many Housing First programs seek to reduce harms for this population, primarily by providing stable housing and tolerating the use of alcohol on-site, which can have intrinsic health and social benefits [9–16]. Housing First programs are associated with decreased use of police and health services, decreased alcohol consumption and problems associated with dependency. Managed alcohol programs (MAPs) take this approach a step further by providing beverage alcohol of known quality to program participants at regular intervals to stabilize drinking patterns and to replace more hazardous nonbeverage alcohol. We are aware of at least ten MAP programs in Canada that offer some form of alcohol administration for those who have been unsuccessful in maintaining housing even when alcohol use is tolerated on-site. The first Canadian MAP, Toronto’s Seaton House, opened in 1997 after an inquiry into three tragic deaths on the streets of Toronto during the winter months. To date, there is limited evidence as to impacts and outcomes of MAPs. Podymow et al. [17] conducted an evaluation of 17 adults involved in the Ottawa MAP and showed improved health outcomes, fewer ED visits, fewer police contacts, and reduced alcohol consumption over an average of 16 months in the MAP. In this paper, we focus on the housing and quality of life outcomes and experiences of participants from one of two pilot studies we have conducted [18, 19].

The research objectives of the pilot evaluation were to establish whether entry into the MAP was associated with (i) significant improvements in health and well-being, (ii) reductions in harms as indicated by decreased use of emergency, hospital, and police services, and (iii) less hazardous patterns of alcohol use as indicated by reduced use of non-beverage alcohol, fewer episodes of severe intoxication, and decreased consumption in high-risk drinking settings without an overall increase in alcohol consumption [20]. In the present paper, we provide a program overview followed by a description of the methodology and findings related to housing and quality of life outcomes as well as insights into the program from the perspectives of MAP participants and staff.

### Program overview

The Kwae Kii Win Centre in Thunder Bay, Ontario, is a 15-bed MAP that opened its doors in February, 2012. The program was established in response to community concerns related to public intoxication and over reliance on police enforcement to address these issues and to meet the needs of men and women experiencing severe and chronic alcohol use problems and homelessness. Criteria for admission to the program include severe alcohol dependence, chronic homelessness, and a high rate of police contacts. The program generally uses 12 % alcohol/volume white wine, and clients may receive up to one 6 oz, i.e., 20.46 mL or 16.14 g of ethanol hourly between the hours of 8 am and 11 pm. To receive a dose, participants must not be overly intoxicated and must have been present at the facility for at least 60 min prior. Drinking outside the program is discouraged, and participants are not allowed to store their alcohol on-site for later consumption.

Residents receive meals, help managing money, access to primary health care, life skills training and counseling, and help accessing legal and income supports. An Elder visits the program weekly and activities such as a drumming circle are offered as part of the shelter program next door. Housing is provided with separate sleeping areas and a number of communal living spaces in the house where the clients can cook, watch television, or do other activities. Residential tenure in the house is contingent on their participation in the MAP. The program is funded in part by contributions from the residents through their social assistance amounts and through a grant to conduct a pilot program. The program is staffed 24 h per day, with at least two staff on at any given time.

### Theoretical framing: structural production of risk

Risk environments are sites or spaces (social or physical) where multiple factors intersect to produce the risk of drug-related harms [21, 22]. The concept of risk environment, as described by Rhodes [22, 23], highlights the social, political, historical, and economic conditions that increase vulnerability to the harms of substance use, particularly illicit drug use. Rhodes (2009) states:

a ‘risk environment’ framework envisages drug harms as a product of the social situations and environments in which individuals participate. It shifts the responsibility for drug harm, and the focus of harm reducing actions, from individuals alone to include the social and political institutions, which have a role in harm production (p. 193).

A social science of harm reduction highlights the influences of micro (physical)- and macro-level environments (social, economic, and policy) in both the production and reduction of drug-related harms [22]. Reducing drug-related harms extends beyond safer use by individuals to a focus on safer settings (physical environments), organizational and governmental policies and practices that shift social, economic, and policy environments. As Rhodes [24] notes, efforts to prevent drug-related harms can be nested within broader programs that reduce social and economic inequities. Housing First programs that integrate harm reduction services, as an example, can address the structural and social production of risk of homelessness and substance use [9, 25–28]. In this paper, we draw on Rhode's risk environment framework to better understand the impact of MAP programs on the lives of participants.

## Methods

We conducted a pilot evaluation of the Thunder Bay MAP using a mixed methods research design that incorporated longitudinal follow-up of MAP participants and controls alongside qualitative interviews. The use of multiple data sources enhances triangulation (convergence of findings) and complementarity (examining different aspects of the same phenomena) to enhance scope and breadth of understanding by including both implementation and outcome assessments [29, 30].

### Sample

All 38 participants (MAP and controls) who responded to the structured survey identified as Indigenous. Of the 18 MAP participants, seven were female and among the 20 controls, eight were female. Mean age of MAP participants was 42 years (range 25–61) and 37 years (range 21–50) for control participants. All resident and control participants were assessed using the Alcohol Use Disorder Identification Test (AUDIT [31] as alcohol dependent and had multiple and repeated experiences with detoxification services. Control participants who met the MAP eligibility requirements were drawn from an emergency homeless shelter. Using purposive sampling, we conducted qualitative interviews with seven MAP residents (three females and four males) who had on average 1 year of experience in the program and four program staff. Residents with at least 1 month of experience in the program, who were willing and able to participate in a qualitative interview, were invited to participate. Very new program participants and those who were unwilling or unable to sit or communicate for 30 min were not interviewed. Program staff had an average of 9 months experience working in the MAP. All participants were recruited through either emails (staff) or referrals from program staff (residents). Ethical approval for the study was obtained from the University of Victoria Research Ethics board, Lakehead University, and the agency where the research was conducted.

### Data collection and analysis

Quantitative structured survey: a structured survey covering the following domains: socio-demographic characteristics; housing status over the past 12 months and housing quality; alcohol and other substance use; severity of alcohol-related problems; and degree of alcohol dependence and quality of life was administered by trained research staff. In this paper, we specifically report on the housing and quality of life data for these participants.

Seven items on the structured interview guide assessed on current housing quality on dimensions such as friendliness and safety [32, 33]. The mean scores on these items were compared between MAP and control participants, and a *t* test was used to determine whether the difference between the two groups was statistically significant. Quality of life was assessed using the WHO-BREF scale [34]. This standardized scale comprises 26 items covering four domains: physical, psychological, relationships, and environments. WHO-BREF domain scores were compared between MAP participants and controls using *t* tests to determine statistically significant differences between the two groups.

### Qualitative data

All the qualitative interviews were conducted by trained qualitative researchers (Gray, Pauly, and Perkin). The resident interviews were conducted on-site by Gray in a private room following an introduction to the research and the researcher. Staff interviewers were conducted primarily over the phone (Pauly or Perkin). Interviews were tape recorded and transcribed verbatim. The interviews were from 30 to 60 min in length, and interviewers used a semi-structured guide consisting of open-ended questions and probes that focused on questions related to understandings of and experiences in the MAP program. Each interview was read and reread by two experienced qualitative researchers (Gray and Pauly) and coded inductively for key ideas and themes that described the experience of being or working in a MAP in relation to housing, health and well-being, and quality of life. For the analysis, we employed constant comparative analysis [35–38]. This method involves detailed coding to develop concepts and relationships among them by comparing incident-to-incident, incident-to-concept, and concept-to-concept to take the analysis from the “ground” up through higher levels of abstraction. An inductive coding framework was developed, and NVivo (NVivo qualitative data analysis software; QSR International Pty Ltd. Version 10, 2012) was used to organize and manage the data. In our interpretation of the data, we drew from risk environment concepts.

## Results

**Fig. 1:**
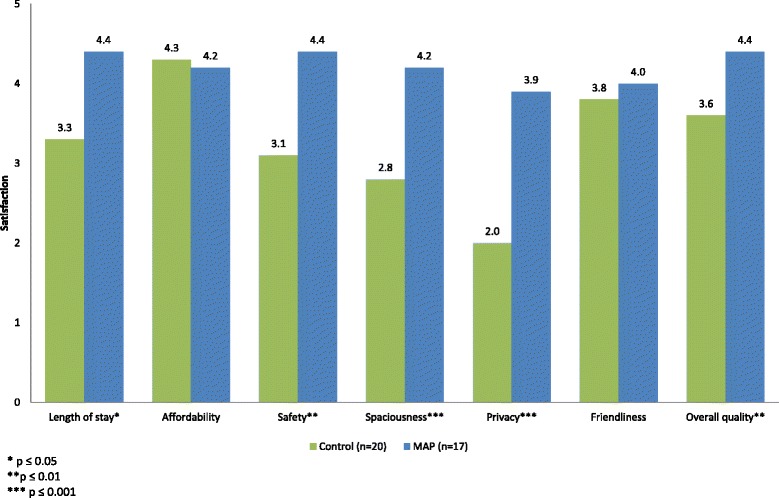
Housing quality and satisfaction. Participants were asked to rate their quality and satisfaction with their housing on seven dimensions using a five-point Likert scale. This figure shows the average scores on each dimension for controls and MAP participants

As per the program criteria, all of the resident participants were homeless (e.g., living outside or staying in an emergency shelter) prior to entering the MAP. Thirteen of the MAP participants retained their housing compared to control participants who remained homeless during the study period. In Fig. 1 below, MAP residents scored statistically significantly higher than controls on the elements of housing quality and satisfaction in length of stay, *t* (25.8) = −2.73, *p* < .05, safety, *t* (28.2) = −3.51, *p* < .01, spaciousness, *t* (30.3) = −3.76, *p* < .001, privacy, *t* (32.8) = −5.23, *p* < .001 and overall quality, *t* (35) = −3.41, *p* < .01.

**Fig. 2:**
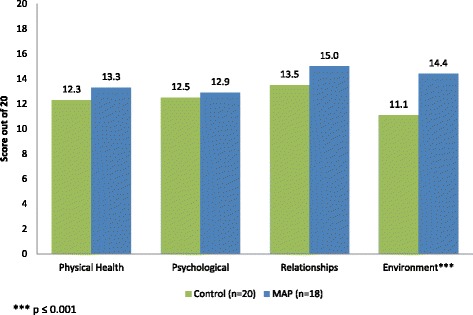
WHO-BREF domains. Figure shows the average scores for controls and MAP participants along the four WHO-BREF dimensions. Scores on this scale can range from 4 to 20

Participants in the MAP rated their psychological well-being, the quality of the environment they lived in and their social relationships higher than controls on the WHO-BREF. The WHO-BREF scores are scaled in a positive direction with higher scores indicating a higher quality of life [34]. As shown in Fig. 2, on each of the four domains, MAP participants (most of whom had been resident in the program longer than 120 days) scored the same or higher than control participants. In the domain of environment, which is an assessment of home life, safety, satisfaction with physical environment, finances, transportation, and access to health services and information, the difference was significant between MAP participants (*M* = 14.37, *SD* = 2.84) and controls (*M* = 11.14, *SD* = 2.78), *t* (36) = −3.54, *p* < .001. Again, safety emerged as significant in the findings.

These findings taken together highlight the importance of MAP as a safer environment compared to pre-MAP environments and the environments of control participants. Below, we present the analysis of the qualitative data that provides a more in-depth description of the role of MAP as a safer microenvironment for participants.

### Before I came here: safer than the streets and shelters

In qualitative interviews, participants explained that prior to moving into the MAP, their life was focused on daily survival to find somewhere to sleep and enough food and alcohol to cope and sustain themselves. For example, one participant stated, “before I came here I wouldn’t care what I was wearing or what I ate….I used to crawl into dumpsters, get something to eat ah… start drinking anything.”. Another participant stated: “I was always out there having to look for it, you know building up some [supply] to make it…to my next drink.” Having to constantly ensure a supply of alcohol can mean more explosive episodes and drinking “anything” including illicit or nonbeverage alcohol. Another participant shares her experience of disconnection and survival on the streets.

I just came back here and……just comin back and liv [ing] on the street… [Alcohol helped to] cover that loneliness I was feelin, because I felt like my family turned their back on me and they don’t want to talk to me, they don’t even attempt to come and see me. I just felt like really alone. Like an outcast. Being on the street with the people I was with, I felt like they took that away you know, that …. outcastness that I felt, you know they accepted me as I was.

This participant identifies, as did others, that having street friends and drinking was a way to cope with lack of meaning, dislocation, loss, trauma, and pain. Like the streets, emergency shelters were identified as unsafe spaces and a site of nonbeverage alcohol use.

Like I couldn’t really sleep when I was there [at a shelter]. People carry knives, there’s fights every night. People are drinking that hairspray and mouthwash, all that other stuff, inside the dorms. But here that doesn’t happen. Yeah, yeah it’s a big difference…Yeah, I felt a lot safer.

All of the participants had multiple and repeated experiences with detoxification and treatment services. One participant explains,

you wanna drink, like I tell my PO too, ‘I don’t wanna go to treatment, I’m gonna end up drinking anyways, it’s not gonna stop me from drinking”, at least I live with the winos that drink every hour and a half [laughs] I think it keeps me like good I guess.

Indeed, many participants we interviewed made reference to the advantage of receiving regular servings of alcohol in the MAP. For example,

It’s making me [not] drink that stuff anymore, and hairspray. Because it- my liver is going, but when I have the wine here, I don’t wake up sick.

Frequently, participants contrasted the safety of the MAP to streets and shelters. One female participant indicated that without the MAP, she would be another statistic among a growing number of Aboriginal women found dead on the street.

My family, they tell me ‘I know you’re still drinking mum, but at least you’re not on the streets, we don’t have to worry about you. We know where we can find you and you know… I wonder how many times …they think that it’s me that’s dead out there when they find a Native woman’.

She highlighted the dangers of being on the street, especially for Aboriginal women, and the safety provided by the program. In participants’ views, both the streets and emergency shelters are high-risk environments that pose dangers to personal safety and perpetuated patterns of unsafe drinking. In contrast, MAP is a safer physical environment.

### Safer than jails and hospitals

Prior to the MAP, if the police apprehended an intoxicated person who did not have housing, the police had no other option than to place that person in a jail cell. Resident and staff participants provided several examples indicating that the MAP provided an alternative to jail. One staff participant commented:

[The police] might pick up one of our residents that’s intoxicated and they’ll call us and ask if they are still in the program and they’ll happily bring them to us.

In this scenario, the person is able to “sleep it off” in their own space under the care of staff. The MAP provided a safer alternative to jail and police enforcement in response to problems of public intoxication.

A key difference noted by participants when comparing MAP to other health-related programs was the extent to which they encountered drug and alcohol-related stigma. One participant describes his experience in healthcare.

I broke my pelvis bone… because …there was ice on the steps there, so, I slipped. They took me to the hospital, then they thought I was just messing around when I told them I couldn’t walk, the doctors thought I just wanted drugs. I was there, maybe a couple days. And they tried to get me to walk, but I couldn’t walk. So I asked the doctor to give me some more tests, so they gave me an MRI, and a CT scan. First they did an X-ray, that’s when they found out that something was wrong. They said “Yeah” so I was in the hospital for a couple of months.

This participant highlights the assumptions that he is “drug seeking” as opposed to being in pain as a result of his injuries. A pervasive and common scenario when people who use substances access healthcare [39]. Another participant explained the questioning he experienced prior to leaving a residential alcohol treatment program for a few hours.

What it meant to me is like they don’t trust me, like over here [MAP] they got a open mind and ‘be safe and come back’, ‘come back in one piece’…What I think is the workers there [residential treatment] they think right away “oh he’s gonna relapse, oh he’s gonna go do something stupid”. So I think that’s why they ask you those questions right off the bat, but here (MAP) they don’t ah ask any questions when you go somewhere. It’s like “I gotta go meet some body; I gotta go do this” “ok bye, come back in one piece, be safe”. It’s almost like they’re giving all their trust in you, the workers here, it’s like they trust you and when I was at the [residential program] one day they did that to me. First thing I thought was after the twenty questions, walk out the door, get on the street, “they don’t trust me, I should just go drink” like ah the [residential program] they’re expecting you to fail. But here they got confidence in you.

This participant highlights the importance of trust and associated feelings of confidence that made him want to stay as opposed to questioning that made him want to leave. In part, the approach described above is a reflection of program goals. One staff participant describes the program goals and measures of success,

There are really only two goals of the program: to lessen the load on the community services, and to provide a better quality of life. That’s it. And so if they still drink hand sanitizer, but their quality of life is better, because now they can go to bed and sleep it off in a safe place, it’s still progress. It’s still better than what it was before. What we’re looking for is incremental success.

The primary goal of the MAP is to provide a safe space while reducing the harms of alcohol use which provides an alternative to the other health and drug-related programs available to participants.

### Finding housing, home, and hope

From the perspective of participants, the MAP was described as being a safer environment with less violence and physical threats to well-being, stigma, and judgments than streets, shelters, jails, and hospitals. The MAP provided a safe refuge from unsafe locations for drinking, violence, and stigma as well as being a source of housing, home, and hope.

### MAP as safe refuge

While MAP participants expressed concerns about lack of respect in other settings, all participants indicated that in the MAP program, people are treated with respect. One resident commented:

I think it’s a pretty good program. If it wasn’t, I would’ve left a long time ago. Here they respect you. And the staff is there all the time. That’s what I like about this place.

One staff member described the program approach as “automatic respect.” She explains:

For anybody to respect me, I have to show them respect, but if I don’t respect a client because oh I don’t like that person he’s just a fall-down-drunk and he’s useless, it’s just a waste of time trying to help him. No, that kind of attitude doesn’t work. And I think for me respect, ….you have to show them that. Because if they don’t see that, it’s not gonna work.

Staff indicated that they started with respect and trust as opposed to residents having to earn respect and trust. Residents stressed the fact that they like the MAP environment because it is respectful and nonjudgmental and characterized by trust and acceptance of who and where they are in their lives. A respectful nonjudgmental approach is a key tenet of harm reduction [40].

The presence of security and staff appeared to enhance residents’ feelings of safety as opposed to feelings of being “under surveillance.” One resident highlighted how initially he did not like the security cameras, but after a period of time, reframed these as contributing to his safety.

Yeah, when I first moved in here, it was like moving into a prison, eh, but a month later I felt a lot safer. And the staff is there all the time. That’s what I like about this place. Yeah, there’s a lot of things I like about this place.

Staff play a key role in creating a safer environment for MAP residents. For example, at the request of residents, staff will screen phone calls and visits from people not in the program.

The staff, like they say, “you wanna go outside [to see your friends]”? I don’t wanna go out …and I know what they are here for, they got some drinks…Yeah, I tell them I don’t wanna go out and [the staff] just say “He’s not in,” or “He’s sleeping”.

As another resident described, staff can act as a referee to reduce conflicts with other residents.

There was a few times that, when a resident was picking on me … and then I had to ask one of the staff to come and sit with me … and then I would tell him I wanted to go to sleep, and he’d stay here until she goes to sleep. So he would stay [with me] until she fell asleep. She doesn’t bother me no more.

Staff contributed to the sense of safety by helping with conflict resolution as well as ensuring safety when drinking and helping to navigate shifts in relationships with street friends.

### MAP as housing and home

Participants described the MAP as a space in which they enact activities of daily life, relearn, and develop community, relationships, and money management skills as well as a place to reconnect with family. For example, one resident described developing a better ability to stand up for herself and communicate with other residents. “I’m learning how to speak up for myself other than just shrink away when, you know, somebody doesn’t… agree with what I’m doing.” In another example, a resident pointed out how they are relearning skills of having a home that were lost when homeless.

The program is… teaching us to be in a home. You know, not like what we’re used to, out on the street. Like re-learning how to be in a house with responsibilities: got to make your bed, do your laundry, sweep, wash the floor, do dishes, and of course, we’re starting to cook. Most of us I think are just re-learning domestic things that you would normally do in a home. It’s another one of the benefits that we get living here.

One staff member noted that MAP residents have many skills coming into the program and that their new learning builds upon their existing knowledge and skills:

I don’t believe that people are starting from ground zero with life-skills. Um, and I just want clarify that we’re not necessarily teaching, quote unquote, life-skills. I think people do have life-skills. They’re different life-skills than ours. I think the notion of life-skills is very North American dominated. You know, that you should learn how to bank, and you should learn how to get up at 8:30 and make your bed, and all that kind of stuff. I mean, I don’t know how to cook moose stew. You know what I mean? But somebody else knows how to prepare wild meat… what we’re doing, I think, is enabling them to use the life-skills that they have.

Most of the participants highlighted that the MAP was not simply “housing” but a “home.” One resident stated, “you feel safe, you feel like you’ve got a warm place to stay, and you know, some home.” Many participants described the MAP residents and staff as being “like family.”

Yeah, we think of each other as a family. When there’s a new person that comes in we welcome them with arms open. And we see they need to be [guided] for the first couple of weeks and we take them and we teach ‘em. And we, ah, show them around and if they need something I’ll show them where to get it, where to ask for it.

They described instances of cooking and eating together in the program as being like a “big family.” Several participants mentioned how residents watch out for each other and generally ensure they are okay. A participant offers a few examples of how he and others “watch over” other residents and provide support.

I wouldn’t let anybody that’s intoxicated get near the oven, that’s one thing that I watch out for. We don’t even let them near the hot plate too. Well if somebody’s hungry and they’re intoxicated, tell them to sit down and wait, we’ll make it for you…[If] they’ve been on a binge again I’ll make something soft like cream of porridge or soup or Jello. Like I know how it feels, I’ve been there, I done it, now I’m tryin hard to stay away from it and it’s been really helpful and the residents here now they see my improvement and they’re actually slowing down on what they used to. Once they see how I’m doing and I guess you could say it opened their eyes too.

A staff person describes the “collective sense” of caring and relationship in the MAP:

When someone gets really sick and has to go to the hospital, or someone is missing, or, whatever, there’s, like, a collective sense of, you know, worry, right? The whole house, kind of, goes into, you know, worry, right? Or concern, or grief, or whatever. So, yeah. ‘Cause they are all very connected as well. Small community, in, in a way, you know?

Moreover, participants also emphasized how the MAP helped to facilitate reunification with their families. Participants described reconnecting with their mothers, fathers, aunts, uncles, children, and grandchildren since coming into the program.

Interviewer: What’s that like to spend time with your family? With your children?

Respondent: Well it feels a lot better now., It wasn’t like that when I was drinking. They look at me and they say oh he’s drunk and let’s go someplace else. Like when they come here they just give me hugs, take pictures, talk.

One participant described how his family was slowly starting to come and visit more now that they were in the MAP as compared to the street.

Ah, I was kinda surprised, but when they see me they’re almost crying, they have a big smile. They see that I have, there’s changes in me like, I ‘m trying hard to change, it doesn’t happen like the snap of a finger, doesn’t take overnight.

This participant went on to describe that instead of seeing their family member being brought home by police and watching them in withdrawal (e.g. witnessing dry heaves, hallucinating, sweating, and pain), “…we go for walks sometimes, we go to the dome, go play mini golf or go to the movies every now and then.” One resident indicated reconnection with family was therapeutic, even though many family members were still “iffy” about them. The reconnection with children and grandchildren was an important milestone:

I see how responsible adults they’ve grown up to be and you know, I missed all that, you know. And I feel sad about it, … at the same time I feel encouraged, like, they never gave up on me.

This participant indicated that the reconnection with children and grandchildren encouraged her to stick with the program. These feelings of home and family as well as reconnecting with family stand in sharp contrast to the sense of loss, disconnection, and isolation that characterized participants’ experiences prior to entering the MAP.

As captured in the narratives and experiences of the participants above, being in MAP provided a sense of hope. As expressed by one participant, there is hope for a better future:

But this program … has given me hope and has allowed me to really think what I wanna do with the rest of my life. And because I was stuck, not stuck, I was I guess you could say rock bottom, you know going home couldn’t get me out of that rock bottom that I was in. But since coming here it’s given me, like I don’t know the word I should use, like ….there’s a horizon waiting for me.

As the following participant highlights, there is hope now for her to reconnect with her Indigenous spiritual and cultural practices. She comments:

I was sick most of the time. Not only alcohol sick but like body sick, spiritually sick. I believe in my culture and my traditions and plus the creator and I lost that you know. I lost that part there where we would you know smudge in the morning and you know and say thank you to our creator and then somehow I just quit doing that. Quit praising, quit praising our creator, I used to be able to, you know, join the celebration, you know there’s pow wows and all that. I don’t even do that anymore you know, put on my regalia and go celebrate. But now I, I haven’t picked it up yet again but it’s like, like I’m slowly getting there. I don’t think you’re ever a whole person because there’s always something new that’s gonna make whole, you know fuller as a person.

For MAP participants, the program provides a safer microenvironment that reduces alcohol-related harms and provides a foundation for reconnecting with family and cultural practices with renewed hope for their future. These findings highlight the importance of a respectful and nonjudgmental space that accepts people and their frailty feelings of family, home, hope and healing are promoted.

## Discussion

There are few studies in which participants with severe alcohol dependence and homelessness have shared their experiences with harm reduction and housing programs. In other research, harm reduction programs for those who inject drugs have been described as providing a refuge from the harms of the street [41] and as a safer environment intervention that alters the structural and environmental context of drug use [42]. In this research, the MAP program can be understood as creating a safer microenvironment that shifts the social, political, and economic conditions of participants with the potential for healing and recovery. Importantly, targeting social conditions that perpetuate harms through the provision of housing and providing alcohol management to directly address environmental conditions faced by homeless persons who are alcohol dependent with multiple failed attempts at treatment may prove more fruitful than a strict focus on individual behavior change or tolerating alcohol use alone [43]. This study helps to extend our understanding of the experiences of persons who are alcohol dependent by demonstrating the complexities involved in the person-environment dynamic and by highlighting the possibilities inherent in building environments that nurture “cultures of resilience to risk” [22] (p. 198).

In Canada, in spite of the lower prevalence of alcohol use among Indigenous populations in general, Indigenous peoples are over-represented among those who are homeless [44–46]. It is well recognized that this is a consequence of colonization and the systematic disadvantaging of and historical trauma experienced by Indigenous people [47–49]. While some authors have suggested that there is a role for harm reduction programs for Indigenous peoples, this is not necessarily a dominant point of view and ongoing substance use may be viewed as incompatible with Indigenous cultural practices [44, 50]. The findings related to respect, feelings of home and family, reconnection with family, and cultural traditions are aligned with First Nations principles for recovery and healing from substance use, particularly the principles of respect and spirit-centered [51]. These findings highlight that regaining a sense of self, home and family, and family reconnection occurred once participants began to stabilize in the MAP. This has profound generational implications for familial relationships as demonstrated by the experiences of reconnecting with children, siblings, and grandchildren. Thus, it is important in future research to more fully investigate MAP participant experiences with MAP as a place of cultural safety, recovery, and healing and to identify recommendations for enhancing cultural safety and the important role of Indigenous culture as treatment [49].

Harm reduction programs are often viewed as controversial because they aim to reduce harms of substance use rather than promote abstinence or reduce substance use. It is evident from these findings that the MAP, as an alcohol harm reduction program, embraced both the philosophy and practice of harm reduction. The emphasis placed on respect, trust, and nonjudgmental care as givens alongside the management of alcohol is consistent with previous research that identified such dimensions as important aspects of a harm reduction approach [52, 53]. Further, the finding that MAPs are not only safer settings but settings where there is a potential for recovery suggests that harm reduction can and should be part of a recovery discourse [54].

Although promising, this study has several limitations. In spite of the inclusion of a control group, a primary limitation of the quantitative data is the small sample size and therefore an inability to generalize these findings. In qualitative interviews, participants primarily contrasted their experiences in MAP with shelters, jails, hospitals, and treatment programs as those were the alternatives available to them. Those eligible for MAP are most often those who have been unsuccessful in retaining housing even when alcohol use is tolerated. Beyond collecting demographic data and being open to learning about potential influences of respondents’ experiences in the MAP, this pilot study did not examine fully the differential impacts of ethnicity and gender on residents’ experiences. We see the need for further research to more fully investigate how culture, ethnicity, and gender as well as related concepts of discrimination and racism influence residents’ experiences of MAPs. We are currently undertaking a national study with a larger sample size and additional qualitative analysis.

## Conclusions

In this study, the MAP provided a reprieve from the street and a shift away from day to day survival, serving as a safer alternative to streets, jails, shelters, and hospitals. The MAP provided a safer physical microenvironment in which the harms of alcohol use were reduced and a harm reduction approach characterized by respect and trust was valued by participants. Evidence for a reduction of alcohol use and related harms for participants on the program is presented in our companion paper (Vallance et al., in press). The MAP environment, characterized by respect, trust, a sense of home, and “feeling like family” while promoting reconnection with family members and the possibility for reconnecting with Indigenous traditions, is consistent with First Nations principles for healing and recovery and principles of harm reduction.
